# Implementation of routinely checked laboratory test results into the national early warning score (NEWS) significantly improves its prognostic ability

**DOI:** 10.1186/2197-425X-3-S1-A334

**Published:** 2015-10-01

**Authors:** A Oskuei, SO Amin, C Daryl, K Gopalratnam, A Geeti, Y Adjepong, SM Hoq, D Kaufman

**Affiliations:** Bridgeport Hospital, Yale University, Bridgeport, United States

## Introduction

Early warning scores such as National Early Warning Score (NEWS) have been used to direct care of deteriorating patients on hospital wards. Certain routine laboratory data including Red Cell Width Distribution (RDW) and renal function have been shown to be independent predictors of mortality.

## Objectives

The purpose of this study was to assess whether addition of RDW and RIFFLE classification, would improve the prognostic ability of the NEWS at the time of rapid response team (RRT) activation.

## Methods

This is an observational study of 198 patients on the hospital wards whose physiological parameters met criteria for RRT activation. Serum Creatinine, eGFR, RDW and NEWS closest to the time of RRT activation were collected from the hospital's electronic medical records. The primary outcomes were in-hospital death, transfer to higher level of care or transition to hospice. The predictive value of NEWS with RIFLE classification, RDW, and all three together were measured and compared using receiver operating characteristic (ROC) curves.

## Results

Out of the 198 RRT activations, 74 patients had at least one positive outcome. For the combined endpoints of in-hospital death, transition to higher level of care or hospice, AU-ROC for NEWS was 0.748, which improved to 0.762 with RDW, and 0.764 with RIFLE classification. AU-ROC for NEWS improved from 0.748 to 0.771 when combined with both RDW and RIFLE classification. Based on NEWS alone, there were 128 patients with moderate and high risk scores, while this number increased to 170 when RDW and RIFLE were added to the tool.

## Conclusions

Addition of RDW and RIFLE classification appears to significantly improve the prognostic performance of NEWS and better identify patients who are at moderate to high risk for adverse events. Further study is needed to assess its implementation in real-time and whether it improves patient outcomes. RDW, serum Creatinine and eGFR are routinely checked during a rapid response event and can be useful in indentifying patients at high risk for poor outcomes.
